# Assessing Food Safety Knowledge and Awareness Among Hospital Food Handlers in Qatar: A Cross-Sectional Study

**DOI:** 10.1155/ijfo/5450277

**Published:** 2025-05-12

**Authors:** Nahlah AlMesbah, Mohamed Aabdien, Latifa AlMohannadi, Iheb Bougmiza

**Affiliations:** ^1^Community Medicine Residency Program - Medical Education, Hamad Medical Corporation, Doha, Qatar; ^2^Community Medicine Residency Program, Primary Healthcare Corporation, Doha, Qatar; ^3^Faculty of Medicine, Sousse University, Sousse Governorate, Tunisia; ^4^College of Medicine, QU Health, Qatar University, Doha, Qatar

**Keywords:** awareness, food handlers, food safety, foodborne diseases, hospitals

## Abstract

In large-scale cooking environments, such as hospital catering, the risk of food contamination due to improper handling escalates with numerous personnel's involvement. Given the critical nature of hospital settings, ensuring stringent adherence to food safety protocols is imperative to prevent adverse health outcomes. This study aims to evaluate food safety knowledge and awareness concerning safe food handling practices among Qatar hospital catering staff, enhancing patient safety and improving healthcare quality. A cross-sectional survey utilizing a validated self-administered questionnaire was conducted among food handlers employed by Hamad Medical Corporation between November 13 and December 7, 2023. The questionnaire was distributed across various hospital facilities. The study involved 366 participants, 60.9% exhibiting good knowledge, 30.1% satisfactory, and 9% lacking adequate food safety knowledge. Similarly, 95.9% of participants exhibited *good*, 3% demonstrated *satisfactory*, and 1.1% displayed *inadequate* awareness regarding safe food handling practices. Significant disparities in total knowledge scores were observed across marital status categories (*p* = 0.043) and job positions (*p* = 0.002). Furthermore, variations in total awareness scores about safe food handling practices were noted among different categories of years of experience (*p* = 0.009) and job positions (*p* = 0.016). Although overall knowledge and awareness among hospital catering staff in Qatar were commendably high, periodic refresher courses are recommended to sustain adherence to best practices. Additionally, continuous oversight and qualitative research to observe actual food handling behaviours are crucial for maintaining optimal food safety standards, complementing self-reported practices.

## 1. Introduction

Food safety is crucial for public health, especially in hospital settings where vulnerable populations are at a higher risk of foodborne illnesses [1, 2]. Ensuring safe and hygienic food prevents health complications and promotes patient well-being. Food handlers play a vital role in maintaining food safety standards, as their knowledge, attitudes, and practices directly impact the quality and safety of food served [3]. To maintain quality healthcare services in Qatar, evaluating and improving the understanding of food safety among the food handlers in the hospitals should be prioritized. Hospitals assist people with weak immune systems; as such, they are likely to be infected by diseases resulting from contaminated food [4].

Food-associated infectious diseases may cause major adverse effects, including longer hospitalization, higher treatment expenses, and death [5]. Some studies have also revealed a lack of awareness regarding food safety and its implementation, indicating the need for further training and education. These include failure to wash hands effectively, poor storage of foods, and poor perception of the crosscontamination risks [6]. These challenges can be addressed by using proper training programs accompanied by evaluation and promotion of food safety culture.

According to statistics from the World Health Organization (WHO), 600 million individuals are affected by foodborne illnesses, and 420,000 people die from these diseases annually. At the same time, 33 million disability-adjusted life years are lost [7]. Stressing food safety and hygiene measures, many scientific papers have reported cases of foodborne diseases, emphasizing the need for proper implementation of standard food safety procedures [8, 9]. In particular, literature has documented foodborne outbreaks linked to inadequate kitchen hygiene practices and poorly managed disease outbreaks in hospital settings [10–14].

For instance, a retrospective review of 50 food poisoning outbreaks from hospitals in Scotland from 1973 to 1977 involving 1530 patients identified *Clostridium perfringens* as the most frequent causal organism, occurring in 31 outbreaks, while *Salmonella* was identified in 11 outbreaks and *Staphylococcus aureus* in three outbreaks, with unidentified etiology in five outbreaks. Noteworthy findings from this review indicated a higher frequency of involvement of psychiatric and geriatric units in these outbreaks [13].

A study examining 48 outbreaks between 1978 and 1987, involving 2287 individuals and 12 fatalities, found a significant decrease in *Clostridium perfringens* cases, but Salmonellosis persisted. The study highlighted the need for improved hospital kitchen protocols, particularly cooking food on the day of consumption, to mitigate outbreaks. Still, crosscontamination from poultry was considered a significant risk [10]. In a separate investigation, a *Clostridium perfringens* food poisoning outbreak affected 38.6% of patients in two hospital wards after consuming roast pork. The source was a local supplier with food safety deficiencies. The oversized meat cuts and lack of equipment for rapidly cooling cooked meat highlighted the hazards of using commercially prepared meats in hospital food service [12].

A review of hospital-acquired listeriosis cases found that 25% were hospital-acquired, with 75% being immunosuppressed. Eight outbreaks showed strong evidence of foodborne transmission within hospitals, linked to food items like sandwiches, butter, and Camembert cheese. The authors recommend hospital policies to enhance food safety, especially by avoiding higher-risk foods for immunocompromised patients [14]. Contamination risks in large-scale cooking operations are high due to multiple individuals involved, posing a significant threat to consumer health and financial implications [15, 16]. Hospital environments, particularly for immunosuppressed, elderly, children, and pregnant women, require strict adherence to hygiene practices, minimum internal cooking temperatures, and appropriate handling and storage of food, including segregation of raw and cooked items, to prevent foodborne illnesses and ensure safety.

Hamad Medical Corporation's (HMC) catering division has obtained a Hazard Analysis Critical Control Point (HACCP) certification in adherence to the International Organization for Standardization 22000 (ISO 22000) food safety management standards. A contracted agency ensures that food handlers receive adequate training and a foundational food safety course. This study aimed to address a research gap in Qatar by assessing food safety knowledge among hospital food handlers. It is crucial as patients with compromised immune systems are at a higher risk of severe health complications from foodborne illnesses. The research, unique in its focus on food handlers in Qatari Hospitals, aims to identify gaps in knowledge and practices, enhancing patient safety and improving healthcare quality. The findings will inform targeted interventions, training programs, and policies in hospital settings.

## 2. Materials and Methods

### 2.1. Study Design

The study used a cross-sectional survey to assess food safety knowledge and awareness among hospital food handlers. The survey was conducted over 4 weeks, from November 13 to December 7, 2023.

### 2.2. Sampling of Respondents

#### 2.2.1. Inclusion Criteria

Inclusion criteria encompassed all personnel within the catering department at HMC who were available during the data collection period. There were no explicit exclusion criteria. Therefore, all food handlers participated entirely.

#### 2.2.2. Sampling Technique

A convenience sampling approach was employed to hand out self-administered questionnaires to participants. Participants received an information sheet outlining the voluntary nature of participation and the option to withdraw by abstaining from returning the questionnaire. Submission of the completed questionnaire was considered consent for study participation. Anonymity was maintained for all completed questionnaires.

#### 2.2.3. Sample Size Calculation

The determined minimum sample size was 366 respondents, calculated using the formula:

 $$\begin{array}{c} n=\frac{Z^{2}\times p\left(1-p\right)}{d^{2}} \end{array}$$

where *n* represents the sample size, *p* signifies the prevalence (39%), *d* indicates the margin of error (5% or 0.05), and *Z* denotes the *Z* statistic for an *α* error of 0.05, corresponding to a 95% confidence level, which equals 1.96.

The prevalence value used in the sample size calculation (39%) was selected based on findings from recent food safety assessments conducted in healthcare and institutional settings within the Middle East region [17–21]. These studies indicate that food safety knowledge deficiencies among food handlers generally range between 33% and 46%. Given this variability, a midpoint estimate of 39% was chosen as a conservative and representative figure for our study population.

Moreover, the selection of this prevalence accounts for institutional variations, ensuring that the sample size is sufficiently powered to detect meaningful differences in knowledge and awareness levels. The decision was made to reflect a realistic estimation of knowledge gaps, balancing underestimation and overestimation, thereby strengthening the study's reliability.

A total of four hospitals were included in this study: Hamad General Hospital, Surgical Specialty Center, Ambulatory Care Center, and Women's Wellness and Research Center. The 366 respondents were proportionally sampled from these hospitals to ensure representation from different healthcare facilities.

This sample size is adequate to detect significant disparities in knowledge and awareness among hospital food handlers, which supports the study's goal of identifying crucial gaps in food safety measures. Furthermore, it enables generalizable findings across the larger hospital food handler population within HMC while combining accuracy with practical data-gathering limits.

### 2.3. Research Instrument

The study utilized a validated questionnaire developed by Teffo and Tabit, demonstrating Cronbach's *α* coefficients ranging from 0.689 to 0.821 [22]. Modifications included the incorporation of “do not know” as a response option and the inclusion of the “>” sign before 74°C for questions about minimum internal cooking temperature, and the addition of the "<" sign before 5°C which was made for questions about receiving and storing food. Adjustments were made due to disparities between correct responses in the questionnaire and those outlined in the Food Safety Code of Practice for Food Services adhered to by food establishments in Qatar [23]. For instance, the correct response for the maximum duration for storing ready-to-eat temperature control for safety (TCS) food was designated as “3 days,” differing from the authors' designation of 7 days. Similarly, “more than 74°C” was the correct answer for questions concerning the minimum internal cooking temperature for eggs and ground beef, deviating from the authors' designation of 68°C for both food items.

As part of their employment requirements at HMC, all food handlers in this study underwent mandatory food safety training conducted by certified food safety professionals from the contracted food service agency. The training curriculum followed international food safety guidelines, including the HACCP principles and ISO 22000 standards, ensuring thorough coverage of essential food safety practices. Course materials comprised interactive lectures, practical demonstrations, and written assessments covering key topics such as proper food handling, hygiene practices, crosscontamination prevention, temperature control, and personal protective equipment usage, all aligned with the Food Safety Code of Practice for Food Services in Qatar, issued by the Ministry of Public Health, to ensure regulatory compliance [23]. Given the diverse linguistic backgrounds of hospital food handlers, the training was offered in multiple languages, including English, Arabic, and Hindi, to enhance accessibility and comprehension. Participants were assessed through written and practical examinations, with periodic refresher courses mandated to ensure continuous adherence to best practices.

### 2.4. Ethics Statement

Before participation, all respondents were given an information sheet detailing the study's objectives, voluntary nature, and confidentiality assurances. Participants consented to the study by completing and returning the questionnaire. They had the right to withdraw by choosing not to submit their responses. Anonymity was strictly maintained for all completed questionnaires. The study was approved by the Institutional Review Board of HMC. Ethical clearance was granted under protocol number MRC-01-23-613. Additionally, permission was obtained from the relevant hospital administrations to survey food handlers.

### 2.5. Data Analysis

Data was analyzed using IBM SPSS Statistics for Windows, Version 26.0 (IBM Corp., Armonk, New York, United States). Quantitative variables were summarised as mean/median and standard deviation (SD)/interquartile range (IQR), while categorical variables were summarised as frequencies and percentages. The knowledge scores were calculated based on the number of correct responses provided by the participants. The total knowledge score ranged from 0 to 13, and participants were classified into three categories: *inadequate knowledge* (0–6), *satisfactory knowledge* (7–9), and *good knowledge* (10–13). Similarly, awareness scores regarding safe food handling practices were derived from responses to awareness-related questions. The total awareness score ranged from 0 to 6 and was categorized as *inadequate* (0–2), *satisfactory* (3–4), and *good* (5–6) [22].

Since the data did not follow a normal distribution, nonparametric statistical tests were employed. The Kruskal–Wallis test assessed score differences across independent variables with three or more categories (age, marital status, education level, and job position). The Mann–Whitney *U* test was employed to compare score differences between independent variables with two categories such as gender and work experience (dichotomized into 10 years or more and less than 10 years). Spearman's correlation test examined the relationships between continuous quantitative variables concerning the total knowledge and awareness scores, including age and years of work experience.

By structuring the knowledge and awareness scores into defined categories, the study ensured a systematic approach to evaluating food handlers' understanding and awareness of food safety. These categorizations allowed for meaningful statistical comparisons and facilitated the identification of areas requiring targeted training interventions.

## 3. Results

### 3.1. Participant Characteristics

Table 1 shows that the study encompassed 366 participants, with an average age of 30.72 years (SD 6.38), ranging from 19 to 54 years. The majority were male (60.9%), single (50.5%), and possessed a high school degree (59.5%). Participants were diverse in their roles, comprising food servers (35.5%), chefs (16.7%), food service supervisors (12%), assistant cooks (6.8%), cooks (6%), food service managers (2.5%), and other support staff (20.5%). Among the support staff (totaling 75), roles included unspecified (24%), receptionists (14.7%), administrative clerks (14.7%), kitchen aides (44%), and miscellaneous roles such as stewards and food safety training coordinators (2.6%). Most participants (47.2%) reported work experience between 6 months and 3 years, while 39% had 6–9 years of experience, and 13.8% had 10 or more years of experience. All participants (99.7%) had taken a food safety course, except one (0.3%) who claimed otherwise.

### 3.2. Knowledge About Receiving and Storing Food

Table 2 presents a summary of participant responses to knowledge-related questions. A significant proportion of participants demonstrated awareness of proper food handling practices, with 71.9% correctly identifying the appropriate temperature (less than 5°C) for receiving TCS foods. Likewise, the majority (70.8%) accurately identified 3 days as the maximum duration for storing ready-to-eat TCS food at less than 5°C.

### 3.3. Knowledge About the Minimum Internal Cooking Temperature Requirement

Most participants (88.3%) demonstrated accurate knowledge of the minimum internal cooking temperature requirements for meat, poultry, and seafood. Similarly, a significant proportion correctly identified the minimum internal cooking temperature requirements for eggs (65.3%) and ground beef (78.4%).

### 3.4. Knowledge About Thawing, Crosscontamination, and Serving

Regarding thawing practices, 65% of participants correctly identified refrigeration as the optimal method for thawing frozen meat, while 22.7% erroneously suggested thawing under running water. Additionally, a majority (87.2%) recognized crosscontamination due to inadequate cleaning and sanitization of food preparation surfaces. Likewise, most participants (76.2%) correctly acknowledged that food handlers should refrain from touching the upper surface of serving plates.

### 3.5. Knowledge About Foodborne Diseases

A substantial portion of participants (82.2%) accurately identified *Salmonella* as the primary cause of foodborne illnesses associated with poultry products. Moreover, most (79.5%) correctly indicated that foodborne bacteria proliferate rapidly at body temperature. Additionally, 88% of participants correctly identified diarrhoea as the predominant symptom of food poisoning.

### 3.6. Knowledge About Vulnerable Groups

Most participants (67.2%) demonstrated awareness that preschool children face heightened susceptibility to foodborne illnesses due to their underdeveloped immune systems. Similarly, the majority (82.5%) correctly identified children, older individuals, and pregnant women as populations at elevated risk of foodborne illnesses compared to the general populace.

### 3.7. Awareness of Safe Food Handling Practices

Nearly all participants (91.8%) concurred that food stored at incorrect temperatures should be promptly discarded. Similarly, a substantial majority (97.8%) agreed on the importance of daily temperature checks for refrigerators, and 98.1% advocated for the segregation of raw and cooked foods during storage.

Table 3 provides a comprehensive overview of participant responses about awareness of safe food handling practices. A large proportion (88.3%) acknowledged the necessity of refraining from food preparation activities during episodes of diarrhoea. Likewise, the majority (96.7%) emphasized the importance of handwashing during food preparation, regardless of others' practices. In comparison, almost all participants (99.2%) recognized the role of improved hygiene in mitigating the risk of foodborne illnesses.

### 3.8. Total Knowledge Score and Associated Factors

Statistically significant differences were observed in the distribution of total knowledge scores across various job positions (*p* = 0.002, *H* = 30.393). Post hoc pairwise comparisons revealed significant disparities in scores between kitchen aides (mean rank = 246.92, median = 11 (IQR 10-12), *n* = 33) and receptionists (mean rank = 119.27, median = 9 (IQR 8-10), *n* = 11, adj.*p* = 0.033), as well as between kitchen aides and food servers (mean rank = 169.97, median = 10 (IQR 8-11), *n* = 130, adj.*p* = 0.011), favouring kitchen aides over the other two groups.

Additionally, a statistically significant difference in knowledge scores was noted based on marital status (*p* = 0.043, *H* = 8.139). Pairwise comparison indicated an important difference between married individuals with a median score of 10.5 (IQR 8-11) and those categorized as “other” with a median score of 6 (adj.*p* = 0.045).

However, the distribution of total knowledge scores did not exhibit significant differences across age categories (*p* = 0.103), gender (*p* = 0.611), level of education (*p* = 0.055), or work experience (*p* = 0.145). Furthermore, Spearman's correlation analysis revealed no significant association between knowledge scores and either age (rho = 0.052, *p* = 0.325) or years of experience (rho = 0.013, *p* = 0.808).  Table 4 summarizes the Kruskal–Wallis test results and Table 5 outlines the Mann–Whitney *U* test results, comparing knowledge scores based on gender and work experience.

**Figure 1:**
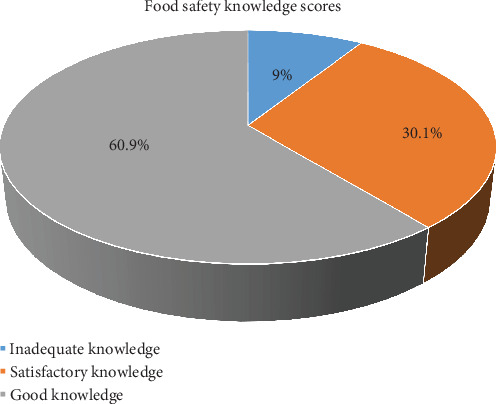
Distribution of knowledge scores among the study participants (*n* = 366).

Figure 1 illustrates the distribution of food safety knowledge scores among study participants. Out of 366 participants, 223 (60.9%) achieved a “*good*” knowledge score, 110 (30.1%) attained a “*satisfactory*” score, and 33 (9.0%) had an “*inadequate*” knowledge score. The calculation of 60.9% was derived by dividing 223 (participants with *good knowledge*) by the total study population of 366 and then multiplying by 100 ((223/366) × 100 = 60.9%).

### 3.9. Total Score for Awareness About Safe Food Handling Practices and Associated Factors

A statistically significant difference was observed in the total score for awareness about safe food handling practices between participants with 10 or more years of work experience (mean rank = 157.49, median = 6 (IQR 5-6), *n* = 49) and those with less than 10 years of work experience (mean rank = 187.52, median = 6 (IQR 6-6), *n* = 317) (*U* = 9041, *z* = 2.608, *p* = 0.009, *r* = 0.1363).

Although a statistically significant difference was initially noted in the scores across job position categories (*p* = 0.016, *H* = 24.68), this significance was lost after adjusting for multiple comparisons using the Bonferroni method. Furthermore, no significant differences were found in total scores for awareness about safe food handling practices across marital status (*p* = 0.311), education level (*p* = 0.301), age (*p* = 0.65), or gender (*p* = 0.26). Moreover, Spearman's correlation analysis revealed no significant association between awareness scores and either age (rho = −0.036; *p* = 0.492) or years of work experience (*rho* = −0.084; *p* = 0.114). Tables 6 and 7 outline the statistical analysis of total awareness scores for safe food handling practices.  Table 6 presents the Mann–Whitney *U* test results based on gender and work experience, and Table 7 summarizes the Kruskal-Wallis results.

**Figure 2:**
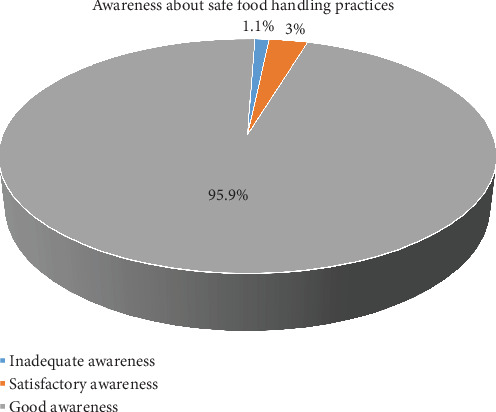
Distribution of scores for awareness of safe food handling practices among the study participants (*n* = 366).

Figure 2 illustrates that most participants, 351 out of 366 (95.9%), achieved a *good* score in awareness about safe food handling practices. In comparison, 11 out of 366 (3%) attained a *satisfactory* score, and only 4 out of 366 (1.1%) received an *inadequate* score. These findings suggest that while awareness is generally high among hospital food handlers, targeted interventions may be necessary for specific subgroups to enhance food safety compliance further.

## 4. Discussion

This study revealed a notable male predominance among participants, contrary to findings in several studies where females typically constitute the majority of food handlers in healthcare and other institutional settings [15, 17, 18, 22]. All food handlers surveyed were non-Qatari, reflecting Qatar's reliance on a diverse migrant workforce in the service industry. This highlights the importance of culturally and linguistically tailored food safety training programs to ensure effective communication and adherence to best practices. Additionally, it is crucial to implement standardized training that accommodates varying levels of prior knowledge and experience among food handlers.

While almost all participants (99.7%) had completed a food safety course, demonstrating a strong commitment to food safety training within Qatar's healthcare food service sector, there remains a need for continuous refresher training. Regular refresher courses, ideally conducted every 6 months to a year, can help reinforce critical food safety principles, update food handlers on new regulations, and correct any misconceptions that may persist.

Regarding specific knowledge areas, our findings highlight strengths and gaps in food safety awareness. For instance, 71.9% of participants correctly identified the appropriate temperature for accepting and storing perishable foods as less than 5°C. While this is an improvement compared to some international studies, it still suggests the need for targeted interventions to improve temperature control practices [22, 24]. Hands-on training and real-time monitoring can be effective strategies to reinforce proper temperature management.

The current study revealed that most participants (70.8%) identified 3 days as the maximum storage duration for ready-to-eat perishable food, which aligns with the official guideline in Qatar and was considered the correct response. In contrast, Teffo and Tabit [22] reported a lower agreement (8.1%) in South Africa, where the official maximum storage duration is 7 days. This discrepancy suggests regional differences in food safety awareness and regulatory standards. While both studies showed a predominant preference for the 3-day limit, our study aligned with official guidelines, whereas the South African study highlighted inconsistencies in public perception [22].

The findings of this study revealed that 65% of participants correctly identified the refrigerator as the appropriate method for thawing frozen meat, indicating a higher level of awareness compared to studies by Elsherbiny et al. [25] in Egypt and Abdelhakeem et al. [17] in Jordan, where only 60.6% and 54.7% of respondents, respectively, recognized this practice. This improvement suggests better access to food safety education or stricter food handling regulations. Additionally, the present study significantly outperformed results from Teffo and Tabit [22] in South Africa and Guennouni et al. [18] in Morocco, where only 31.9% and 28% of participants, respectively, answered correctly, likely reflecting differences in food safety training, socioeconomic factors, or cultural practices. However, 22.7% of participants believed that thawing at room temperature was acceptable, highlighting a persistent knowledge gap. While this percentage is much lower than the 72.8% reported by Bou-Mitri et al. [20] in Lebanon, it still underscores the need for targeted interventions such as practical demonstrations and routine audits to reinforce proper thawing practices and reduce the risk of foodborne illnesses.

In addition, the present study found that 88.3% of participants correctly identified the minimum internal cooking temperature for meats, poultry, and seafood, highlighting strong awareness but a need for reinforcement through visual aids and exercises persists. In contrast, awareness was much lower in South Africa (9.05%), Egypt (35.6%), and Saudi Arabia (49.8%), as reported by previous studies [19, 22, 25]. These variations may arise from differences in food safety education, cultural practices, and resource availability. The findings emphasize the importance of targeted interventions like educational campaigns and hands-on training. Access to temperature measurement tools can improve food safety awareness and reduce health risks.

Regarding foodborne disease awareness, 87.2% of participants identified *Salmonella* as the leading cause of foodborne illness, 79.5% recognized that bacteria double rapidly at 37°C, and 88% knew diarrhoea is the most common symptom of food poisoning. In contrast, the South African study by Teffo and Tabit [22] reported much lower awareness, with only 47.1% recognizing *Salmonella* and 38.1% understanding bacterial growth at 37°C. However, 91.9% of participants in the South African study correctly identified diarrhoea as a key symptom, slightly higher than the 88% in the present study. These differences indicate a stronger overall food safety awareness in the present study, except for symptom recognition, where both studies showed similar knowledge. The findings highlight the need for region-specific food safety education to address varying knowledge gaps [22].

Regarding high-risk populations, 67.2% of participants correctly identified preschool children as high-risk due to their immunocompromised status. In comparison, 82.5% accurately recognized vulnerable populations prone to foodborne illnesses, highlighting a strong awareness of at-risk groups. However, further emphasis is needed on pregnant women and the elderly to ensure comprehensive understanding among food handlers. In comparison, the South African study by Teffo and Tabit [22] reported slightly lower awareness, with 66.7% recognizing preschool children as at-risk and 71.4% correctly identifying high-risk groups for foodborne illnesses. Additionally, Al Banna et al. [24] found that 76.4% of Bangladeshi participants had poor knowledge of the issue, with many incorrectly assuming all people, including children, elderly, and pregnant women, were equally vulnerable. These findings indicate that the present study's participants demonstrated greater awareness of foodborne illness risks compared to other studies.

The distribution of total food safety knowledge scores differed across the various job positions which is consistent with the findings from the Egyptian study by Elsherbiny et al. [25], and the Turkish research by Ulusoy and Çolakoğlu [26] but inconsistent with the South African study by Teffo and Tabit [22]. This was expected as the people with more responsibilities would show higher scores because of their daily practice.

The “other” and married groups had significantly different distributions of overall food safety knowledge ratings, with the latter scoring higher. While statistically significant, caution should be taken in interpreting this difference due to the small sample size (three in the “other” group), which may not accurately represent the population. More research in studies with a wider subgroup sampling is needed to corroborate these findings.

In the present study and the study done by Teffo and Tabit [22], no effect was demonstrated between the level of educational attainment and the overall score of total food safety knowledge, as we had expected. This is contrary to the results obtained from other research conducted by Abdelhakeem et al. [17] and Akabanda et al. [15].

Our study found no significant associations between total food safety knowledge scores and age or work experience, emphasizing the need for structured learning interventions like competency-based assessments, mentorship programs, and interactive workshops to bridge knowledge gaps. In contrast, Elsherbiny et al. [25] in Egypt reported that longer work experience and older age were linked to higher knowledge scores, suggesting that expertise enhances food safety knowledge. Similarly, Bou-Mitri et al. [20] in Lebanon found that participants with 21 or more years of work experience demonstrated superior knowledge. These findings indicate that while work experience may play a role in knowledge acquisition, structured training remains essential for all demographics.

The distribution of total food safety knowledge scores did not differ by gender in our study, consistent with the Turkish research by Ulusoy and Çolakoğlu [26]. This aligns with expectations considering the mandatory nature of food safety courses for all participants.

Participants in our study demonstrated better awareness of food safety practices than those in the South African study by Teffo and Tabit [22]. For instance, a higher proportion of participants in our study (98.1%) emphasized the importance of keeping raw and cooked food separate, contrasting with rates of 40.9%, 80.6%, 87.6%, 90%, and 92.7% in the Egyptian study by Elsherbiny et al. [25], Sri Lankan study by Adikari et al. [27], South African study by Teffo and Tabit [22], Moroccan study by Guennouni et al. [18], and Bangladeshi study by Al Banna et al. [24], respectively.

However, concerning food handling during episodes of diarrhoea, our study revealed that 8.7% of participants did not agree with avoiding food preparation for others, with an additional 3% expressing uncertainty on the matter. This highlights a critical area requiring attention, as outlined in the Food Safety Code of Practice for Food Services, emphasizing the importance of food handlers' awareness of health and hygiene obligations, including reporting any health conditions to supervisors and abstaining from food handling when a contamination risk exists [23]. Such discrepancies may stem from concerns about reporting illness due to potential consequences, particularly as food handlers are contracted through a third-party employer. Furthermore, additional training is warranted to ensure correct practices when experiencing diarrhoea.

The current study revealed that the comparatively young employees with experience of not more than 10 years had a better attitude towards safe food handling practices than their older employees with 10 years or more experience. This could be attributable to training that was still recent and, therefore, the “novelty effect” regarding routine tasks and the workplace environment. Besides that, different generations' working motivations and various learning styles can define this difference where new employees are motivated to apply their knowledge and be promoted.

Our findings indicate that while food safety knowledge among healthcare food handlers in Qatar is relatively strong, there is room for improvement in specific areas. Regular refresher training, hands-on demonstrations, and continuous monitoring should be integrated into food safety programs to enhance compliance with food safety protocols. In addition, employing multilingual training materials and practical assessments will ensure all food handlers, regardless of educational or cultural background, can fully grasp and apply critical food safety principles.

## 5. Strengths and Limitations

The study marks the inaugural investigation into hospital food handlers' understanding of food safety in Qatar, emphasizing HMC personnel. A “do not know” option has been added to the questionnaire to reduce speculative answers and potential response bias. Limitations include the questionnaire's self-administered format as participants are unlikely to seek clarifications for questions they do not understand.

## 6. Conclusion

The study found that hospital food handlers strongly understand safe food handling practices, including proper cooking temperatures and crosscontamination risks in Qatar. However, there are variations in knowledge based on job roles and marital status, as well as variations in safe food handling practices based on work experience. The study suggests that while overall food safety awareness is high, there are areas where training and consistency could be enhanced. The study indicates that a comprehensive and standardized food safety training program provided by HMC as a precondition for employment would ensure up-to-date knowledge of best practices. This program should include detailed modules on hazard analysis, hygiene protocols, and emergency response measures. It should be recurrent, tied to contract renewals, and continually revised to align with the latest Food Safety Code of Practice for Food Services. Future research should explore the effectiveness of such training programs in improving compliance and reducing foodborne illness incidents. Additionally, studies could investigate the role of cultural and demographic factors in shaping food safety behaviours among hospital food handlers to tailor interventions more effectively.

## Figures and Tables

**Table 1:** Population characteristics (*n* = 366).

**Variables**	**Frequency (%)**
Age	
19–25 years old	84 (23.3%)
26–35 years old	201 (55.7%)
36–45 years old	70 (19.4%)
46–54 years old	6 (1.7%)
Years of experience	
6 months–3 years	168 (47.2%)
4–6 years	108 (30.3%)
7–9 years	31 (8.7%)
10–19 years	38 (10.7%)
20–28 years	11 (3.1%)
Gender	
Male	223 (60.9%)
Female	143 (39.1%)
Marital status	
Single	185 (50.5%)
Married	172 (47%)
Divorced	6 (1.6%)
Widowed	0 (0%)
Other	3 (0.8%)
Level of education	
High school degree	217 (59.5%)
Bachelor's degree	88 (24.1%)
Postgraduate degree	38 (10.4%)
No high school degree	22 (6%)
Current position	
Manager	9 (2.5%)
Supervisor	44 (12%)
Chef	61 (16.7%)
Cook	22 (6%)
Assistant cook	25 (6.8%)
Food server	130 (35.5%)
Other support staff	75 (20.5%)
Have taken a food safety course	
Yes	365 (99.7%)
No	1 (0.3%)

**Table 2:** Responses to knowledge questions about receiving, storing, cooking, thawing, and vulnerable groups (*n* = 366).

**Responses**	**Frequency (%)**
Knowledge regarding receiving and storing food	
Temperature for receiving perishable food items	
≤ 0°C	11 (3%)
< 5°C	263 (71.9%)
≤ 7°C	57 (15.6%)
≤ 10°C	14 (3.8%)
Do not know	21 (5.7%)
Maximum duration for storing prepared perishable food items at < 5°C	
3 days	259 (70.8%)
5 days	60 (16.4%)
7 days	31 (8.5%)
9 days	0 (0%)
Do not know	16 (4.4%)

Knowledge regarding minimum internal cooking temperature	
For meat, poultry, and seafood	
57°C	0 (0%)
63°C	16 (4.4%)
68°C	11 (3%)
> 74°C	323 (88.3%)
Do not know	16 (4.4%)
For eggs that will be hot-held	
57°C	7 (1.9%)
63°C	50 (13.7%)
68°C	49 (13.4%)
> 74°C	239 (65.3%)
Do not know	20 (5.5%)
For ground beef	
57°C	9 (2.5%)
63°C	24 (6.6%)
68°C	21 (5.7%)
> 74°C	287 (78.4%)
Do not know	25 (6.8%)

Knowledge regarding thawing, cross-contamination, and serving	
The best way to thaw frozen meat	
At room temperature	83 (22.7%)
In the refrigerator	238 (65%)
Under cold water	33 (9%)
Using the microwave oven	2 (0.5%)
Do not know	10 (2.7%)
Not cleaning or sanitizing a food preparation table between uses will result in	
Unpleasant flavors in food	13 (3.6%)
Cross-contamination	319 (87.2%)
Toxic metal poisoning	17 (4.6%)
Time-temperature abuse	3 (0.8%)
Do not know	14 (3.8%)
Section of a serving plate to avoid touching	
Bottom	44 (12%)
Edge	23 (6.3%)
Side	14 (3.8%)
Top	279 (76.2%)
Do not know	6 (1.6%)

Knowledge regarding foodborne diseases	
The main cause of foodborne disease in poultry products	
*Salmonella enterica*	301 (82.2%)
*Staphylococcus aureus*	26 (7.1%)
*Escherichia coli*	3 (0.8%)
*Clostridium botulinum*	7 (1.9%)
Do not know	29 (7.9%)
Food poisoning bacteria, at body temperature, will	
Die	16 (4.4%)
Do not grow	18 (4.9%)
Grow quickly	291 (79.5%)
Grow slowly	24 (6.6%)
Do not know	17 (4.6%)
The most common symptom of food poisoning	
Headache	19 (5.2%)
Diarrhea	322 (88%)
Rash	6 (1.6%)
Constipation	4 (1.1%)
Do not know	15 (4.1%)

Knowledge regarding vulnerable groups	
Reason preschool children are at higher risk of foodborne illnesses	
Preschool children have not built up strong immune systems	246 (67.2%)
Preschool children are more likely to spend time in hospital	3 (0.8%)
Preschool children are more likely to suffer allergic reactions	11 (3%)
All of the above	97 (26.5%)
Do not know	9 (2.5%)
The group most vulnerable to foodborne illnesses	
Children	27 (7.4%)
Older people	17 (4.6%)
Pregnant women	11 (3%)
All of the above	302 (82.5%)
Do not know	9 (2.5%)

**Table 3:** Responses to questions assessing awareness about safe food handling practices (*n* = 366).

**Responses**	**Frequency (%)**
Food stored at an incorrect temperature must always be discarded	
Agree	336 (91.8%)
Disagree	26 (7.1%)
Not sure	4 (1.1%)
The temperature of refrigerators should be checked at least once per day	
Agree	358 (97.8%)
Disagree	7 (1.9%)
Not sure	1 (0.3%)
Raw and cooked food should be separated during storage	
Agree	359 (98.1%)
Disagree	3 (0.8%)
Not sure	4 (1.1%)
When ill with diarrhoea, food handlers should avoid partaking in food preparation	
Agree	323 (88.3%)
Disagree	32 (8.7%)
Not sure	11 (3%)
Washing hands during food preparation is important even if others do not wash theirs	
Agree	354 (96.7%)
Disagree	10 (2.7%)
Not sure	2 (0.5%)
Improving hygiene practices reduces the risk of foodborne illnesses	
Agree	363 (99.2%)
Disagree	2 (0.5%)
Not sure	1 (0.3%)

**Table 4:** Kruskal–Wallis results of association between the sociodemographic factors and the total knowledge score.

**Sociodemographic factor**	**Frequency**	**H** ** statistic**	**Degrees of freedom**	**p** ** value**
Age	366	6.183	3	0.103
Level of education	365	7.586	3	0.055
Job position	366	30.393	12	**0.002**
Marital status	366	8.139	3	**0.043**

*Note: p* values that are bold indicate that the results are statistically significant (below 0.05).

**Table 5:** Mann–Whitney *U* results of association between sociodemographic factors and the total knowledge score.

**Sociodemographic factor**	**Sample 1**	**Mean rank**	**Median (IQR)**	**Sample 2**	**Mean rank**	**Median (IQR)**	**p** ** value**
Gender	Male (*n* = 223)	185.71	10 (8–11)	Female (*n* = 143)	180.05	10 (8–11)	0.611
Work experience	10 years or more (*n* = 49)	203.67	11 (9–11)	Less than 10 years (*n* = 317)	180.38	10 (8–11)	0.145

**Table 6:** Mann–Whitney *U* results of association between sociodemographic factors and the total score for awareness about safe food handling practices.

**Sociodemographic factor**	**Sample 1**	**Mean rank**	**Median (IQR)**	**Sample 2**	**Mean rank**	**Median (IQR)**	**p** ** value**
Gender	Male (*n* = 223)	179.96	6 (6–6)	Female (n=143)	189.02	6 (6–6)	0.260
Work experience	10 years or more (*n* = 49)	157.49	6 (5–6)	Less than 10 years (*n* = 317)	187.52	6 (6–6)	**0.009**

*Note: p* values that are bold indicate that the results are statistically significant (below 0.05).

**Table 7:** Kruskal–Wallis results of association between the sociodemographic factors and the total score for awareness about safe food handling practices.

**Sociodemographic factor**	**Frequency**	**H** ** statistic**	**Degrees of freedom**	**p** ** value**
Age	366	1.641	3	0.650
Level of education	365	3.653	3	0.301
Job position	366	24.68	12	**0.016**
Marital status	366	3.579	3	0.311

*Note:p* values that are bold indicate that the results are statistically significant (below 0.05).

## Data Availability

All the data used and analyzed are present within the manuscript.

## Nomenclature

HACCP: Hazard analysis critical control point
HMC: Hamad Medical Corporation
IQR: Interquartile range
ISO 22000: International Organization for Standardization for Dealing with Food Safety 22000
SD: Standard deviation
TCS: Temperature control for safety
WHO: World Health Organization

## References

[1] Lund B. M. (2015). Microbiological Food Safety for Vulnerable People. *International Journal of Environmental Research and Public Health*.

[2] Mensah P., Mwamakamba L., Mohamed C., Nsue-Milang D. (2012). Public Health and Food Safety in the WHO African Region. *African Journal of Food, Agriculture, Nutrition and Development*.

[3] Faour-Klingbeil D. (2022). Food Safety Knowledge, Attitudes, and Practices Among Food Handlers in Foodservice Establishments in the Arab Countries of the Middle East. *Food Safety in the Middle East*.

[4] Lund B. M. (2019). Provision of Microbiologically Safe Food for Vulnerable People in Hospitals, Care Homes and in the Community. *Food Control*.

[5] Hoffman S., Maculloch B., Batz M. (2015). *Economic Burden of Major Foodborne Illnesses Acquired in the United States*.

[6] Amegah K. E., Addo H. O., Ashinyo M. E. (2020). Determinants of Hand Hygiene Practice at Critical Times Among Food Handlers in Educational Institutions of the Sagnarigu Municipality of Ghana: A Cross-Sectional Study. *Environmental Health Insights*.

[7] WHO (2022). Food Safety. https://www.who.int/news-room/fact-sheets/detail/food-safety.

[8] Giannatale E. D., Sacchini L., Persiani T., Alessiani A., Marotta F., Zilli K. (2012). First Outbreak of Food Poisoning Caused by Salmonella enterica Subspecies enterica serovar Berta in Italy. *Letters in Applied Microbiology*.

[9] Marineli F., Tsoucalas G., Karamanou M., Androutsos G. (2013). Mary Mallon (1869-1938) and the History of Typhoid Fever. *Annals of Gastroenterology*.

[10] Collier P. W., Sharp J. C. M., MacLeod A. F., Forbes G. I., Mackay F. (1988). Food Poisoning in Hospitals in Scotland, 1978-87. *Epidemiology and Infection*.

[11] Karabela Ş. N., Şenoğlu S., Aydin Ö. A., Baydili K. N., Aksu Ö., Yaşar K. K. (2022). Foodborne Streptococcal Tonsillopharyngitis Outbreak in a Hospital. *European Journal of Public Health*.

[12] Regan C. M., Syed Q., Tunstall P. J. (1995). A Hospital Outbreak of Clostridium Perfringens Food Poisoning–Implications for Food Hygiene Review in Hospitals. *Journal of Hospital Infection*.

[13] Sharp J. C., Collier P. W., Gilbert R. J. (1979). Food Poisoning in Hospitals in Scotland. *Epidemiology & Infection*.

[14] Silk B. J., McCoy M. H., Iwamoto M., Griffin P. M. (2014). Foodborne Listeriosis Acquired in Hospitals. *Clinical Infectious Diseases*.

[15] Akabanda F., Hlortsi E. H., Owusu-Kwarteng J. (2017). Food Safety Knowledge, Attitudes and Practices of Institutional Food-Handlers in Ghana. *BMC Public Health*.

[16] Annor G. A., Baiden E. A. (2011). Evaluation of Food Hygiene Knowledge Attitudes and Practices of Food Handlers in Food Businesses in Accra, Ghana. *Food and Nutrition Sciences*.

[17] Abdelhakeem A. A., Feyza B., Hekmat A.-A. (2021). Food Safety Knowledge Among Food Handlers in Hospitals of Jordan. *Food Science and Technology*.

[18] Guennouni M., Admou B., Bourrhouat A. (2022). Knowledge and Practices of Food Safety Among Health Care Professionals and Handlers Working in the Kitchen of a Moroccan University Hospital. *Journal of Food Protection*.

[19] Alsultan S. B., Allowaymi S. S., Alshammari G. M. (2023). Cross-Sectional Investigation of the Awareness and Practices of Food Safety Among Food Service Catering Staff in Riyadh City Hospitals During the Coronavirus Pandemic. *Healthcare*.

[20] Bou-Mitri C., Mahmoud D., El Gerges N., Abou Jaoude M. (2018). Food Safety Knowledge, Attitudes and Practices of Food Handlers in Lebanese Hospitals: A Cross-Sectional Study. *Food Control*.

[21] Aljasir S. F. (2023). Food Safety Knowledge and Practices Among Food Handlers and Consumers in Gulf Countries: An Integrative Review. *Global Public Health*.

[22] Teffo L. A., Tabit F. T. (2020). An Assessment of the Food Safety Knowledge and Attitudes of Food Handlers in Hospitals. *BMC Public Health*.

[23] Ministry of Public Health Qatar (2020). *Food Safety Code of Practice for Food Services - Food Service Establishment Guidelines*.

[24] Al Banna M. H., Khan M. S. I., Rezyona H. (2022). Assessment of Food Safety Knowledge, Attitudes and Practices of Food Service Staff in Bangladeshi Hospitals: A Cross-Sectional Study. *Nutrients*.

[25] Elsherbiny N. M., Sobhy S. A., Fiala L., Abbas M. A. (2019). Knowledge, Attitude and Practices of Food Safety Among Food Handlers in Ismailia City Hospitals, Egypt. *International Journal of Advanced Community Medicine*.

[26] Ulusoy B. H., Çolakoğlu N. (2018). What Do They Know About Food Safety? A Questionnaire Survey on Food Safety Knowledge of Kitchen Employees in Istanbul. *Food and Health*.

[27] Adikari A. M. N. T., Rizana M. S. F., Amarasekara T. P. (2016). Food Safety Practices in a Teaching Hospital in Sri Lanka. *Procedia Food Science*.

